# Dss1 Is a 26S Proteasome Ubiquitin Receptor

**DOI:** 10.1016/j.molcel.2014.09.008

**Published:** 2014-11-06

**Authors:** Konstantinos Paraskevopoulos, Franziska Kriegenburg, Michael H. Tatham, Heike I. Rösner, Bethan Medina, Ida B. Larsen, Rikke Brandstrup, Kevin G. Hardwick, Ronald T. Hay, Birthe B. Kragelund, Rasmus Hartmann-Petersen, Colin Gordon

**Affiliations:** 1 Medical Research Council Human Genetics Unit, Western General Hospital, Crewe Road, Edinburgh EH4 2XU, Scotland, UK; 2 Department of Biology, University of Copenhagen, Ole Maaløes Vej 5, 2200 Copenhagen N, Denmark; 3 Centre for Gene Regulation and Expression, College of Life Sciences, University of Dundee, Dundee DD1 5EH, Scotland, UK; 4 Wellcome Trust Centre for Cell Biology, University of Edinburgh, Edinburgh, EH9 3JR, Scotland, UK

## Abstract

The ubiquitin-proteasome system is the major pathway for protein degradation in eukaryotic cells. Proteins to be degraded are conjugated to ubiquitin chains that act as recognition signals for the 26S proteasome. The proteasome subunits Rpn10 and Rpn13 are known to bind ubiquitin, but genetic and biochemical data suggest the existence of at least one other substrate receptor. Here, we show that the phylogenetically conserved proteasome subunit Dss1 (Sem1) binds ubiquitin chains linked by K63 and K48. Atomic resolution data show that Dss1 is disordered and binds ubiquitin by binding sites characterized by acidic and hydrophobic residues. The complementary binding region in ubiquitin is composed of a hydrophobic patch formed by I13, I44, and L69 flanked by two basic regions. Mutations in the ubiquitin-binding site of Dss1 cause growth defects and accumulation of ubiquitylated proteins.

## Highlights

• Dss1 is a ubiquitin-binding protein
• Dss1 binds ubiquitin via an intrinsically disordered region
• The ubiquitin-binding activity of Dss1 is required for function

## Graphical Abstract

**Figure:**
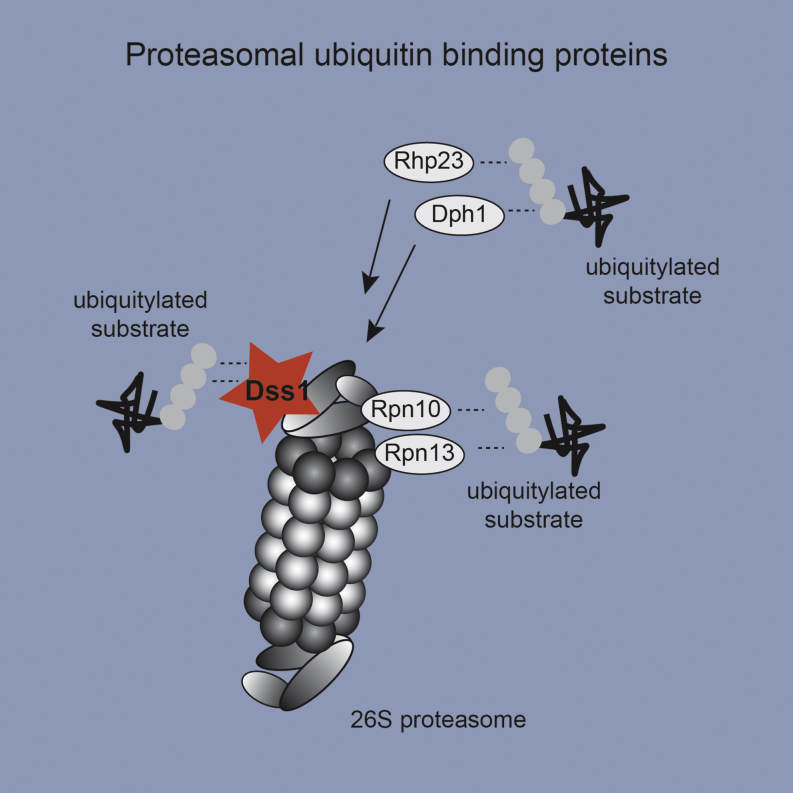

## Teaser

Paraskevopoulos et al. demonstrate that Dss1 is a ubiquitin receptor for the 26S proteasome that interacts with ubiquitin via ubiquitin-binding sites located in an intrinsically disordered region. Mutation of the ubiquitin-binding sites causes growth defects and accumulation of ubiquitylated proteins.

## Introduction

The ubiquitin-proteasome system (UPS) is the major pathway for protein degradation in eukaryotic cells, regulating most cellular processes, including cell division, signal transduction, and development (Finley, 2009). Before degradation, proteins are conjugated to ubiquitin chains that act as recognition signals for the 26S proteasome, a large proteolytic complex that degrades substrate proteins (Finley, 2009).

Although proteasome function has been extensively studied, our knowledge of how this particle recognizes ubiquitylated substrates remains incomplete. Since the identification of the first intrinsic proteasomal ubiquitin receptor, Rpn10, studies have identified a group of so-called UBL-UBA domain proteins that act as transient, extrinsic proteasome substrate receptors (Deveraux et al., 1994, Seeger et al., 2003, Su and Lau, 2009, Wilkinson et al., 2001). More recently, an additional novel intrinsic receptor, Rpn13, was identified (Husnjak et al., 2008, Schreiner et al., 2008). However, budding yeast cells, deleted for the UBL-UBA domain proteins and mutated in both the Rpn10 and Rpn13 ubiquitin-interacting regions, are still viable (Husnjak et al., 2008). Moreover, ubiquitin conjugates still bind to 26S proteasomes lacking the ubiquitin-interacting regions of Rpn10 and Rpn13 (Peth et al., 2010). As proteasome function is essential, at least one additional ubiquitin receptor remains to be discovered (Saeki and Tanaka, 2008). Here, we present structural, biochemical, and genetic data that the disordered and multifunctional protein Dss1 (known as Sem1 in budding yeast), is another ubiquitin-binding subunit of the 26S proteasome.

## Results

### Ubiquitin Binding to Rpn10 Is Not Essential for Viability

**Figure 1:**
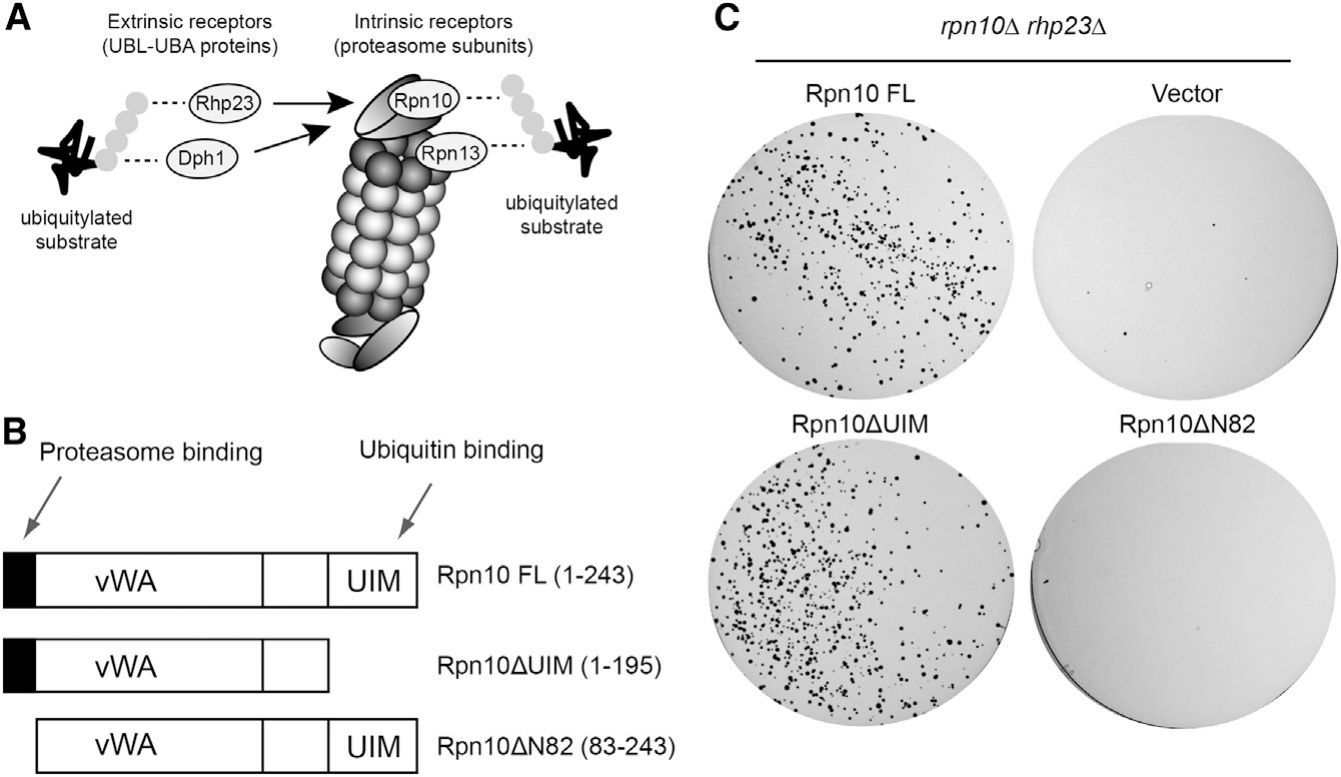
The Rpn10 UIM Is Not Responsible for the *rhp23*Δ*rpn10*Δ Synthetic Lethality (A) Substrate recognition by the 26S proteasome is mediated via intrinsic receptors that are subunits (Rpn10 and Rpn13) and extrinsic receptors that are UBL-UBA domain cofactors (Rhp23/Rad23 and Dph1/Dsk2). The substrate is depicted as a black thread, and ubiquitin is depicted as a gray sphere. (B) The domain organization of full-length (FL) Rpn10, Rpn10ΔUIM (deleted of the UIM domain to abolish ubiquitin binding), and Rpn10ΔN82 (82-residue N-terminal deletion to abolish proteasome binding) (Seeger et al., 2003). (C) In vivo assay of *rpn10*Δ and *rhp23*Δ deletion strains transformed to express the indicated constructs. The two strains were crossed to generate a double deletion. Following crossing, 10,000 spores were plated on media that selected for both deletion mutants and the expression vector. See also Figure S1.

In fission yeast, substrate recognition by the 26S proteasome is accomplished by two intrinsic proteasome subunits, Rpn10 and Rpn13, and two extrinsic UBL-UBA domain proteasome cofactors, Rhp23 and Dph1 (Finley, 2009, Hartmann-Petersen et al., 2003, Sakata et al., 2012, Wilkinson et al., 2001) (Figure 1A). Studies have shown these receptors to be functionally redundant (Husnjak et al., 2008, Peth et al., 2010, Wilkinson et al., 2001). It was previously demonstrated, both in budding and fission yeast, that the gene for the UBL-UBA domain protein Rad23 (Rhp23 in fission yeast) functionally overlapped with the gene encoding the 26S proteasome ubiquitin receptor subunit Rpn10. Specifically, only a double deletion mutant (*rpn10*Δ*rhp23*Δ) displayed severe growth defects (Wilkinson et al., 2001). In addition, Rhp23 variants unable to bind ubiquitin or the proteasome could not rescue the growth defects of the double mutant, implying that substrate recognition was at least partly responsible for the observed phenotypes (Wilkinson et al., 2001). Therefore, we asked whether lack of the ubiquitin- or proteasome-binding functions of Rpn10 contribute to the severe phenotype of the *rpn10*Δ*rhp23*Δ double mutant. To this end, we cloned constructs of *rpn10* that lacked the ubiquitin interaction motif (UIM), Rpn10ΔUIM, or the N-terminal proteasome-binding region, Rpn10ΔΝ82 (Figure 1B) (Seeger et al., 2003). The constructs were integrated into both *rpn10*Δ and *rhp23*Δ strains. These strains were then crossed, and the ability of the Rpn10 constructs to rescue the growth defects of the *rpn10*Δ*rhp23*Δ double mutant were assayed by plating and selecting for the relevant spores. Surprisingly, this revealed that the Rpn10ΔUIM construct rescued the growth defects as efficiently as the full-length construct (Figure 1C; Figure S1A available online), while the Rpn10ΔΝ82 proteasome-binding mutant did not (Figure 1C). This implies that loss of Rpn10 ubiquitin binding does not contribute to the severe phenotype of the *rpn10*Δ*rhp23*Δ double mutant.

The fact that the *rhp23*Δ*rpn10*ΔUIM mutant is viable is consistent with previous work, suggesting that the vWA domain has some unknown facilitator function in the UPS (Mayor et al., 2007, Peth et al., 2010, Verma et al., 2004) and shows that other proteasomal substrate receptors functionally overlap with Rpn10 and Rhp23. Currently, the remaining known receptors and shuttle proteins are the UBL-UBA protein Dsk2 (Dph1 in fission yeast) and Rpn13 (Rpn13a and Rpn13b in fission yeast) that associate with both ubiquitin and the proteasome. To test these candidates genetically, null mutants were constructed for each and subsequently crossed to create the appropriate genetic backgrounds. We postulated that, if either of these receptors functionally overlapped with Rpn10 and Rad23, then deletion of its gene in the *rpn10*Δ*rhp23*Δ background should prevent rescue of the *rpn10*Δ*rhp23*Δ phenotype by the Rpn10ΔUIM construct. Surprisingly, the Rpn10ΔUIM construct once again rescued the *dph1*Δ*rpn10*Δ*rhp23*Δ triple (Figure S1B) and *rpn13a*Δ*rpn13b*Δ*rpn10*Δ*rhp23*Δ quadruple deletion mutants (Figure S1C). This implies that neither Dph1 nor Rpn13 were responsible for the rescue of the *rpn10*Δ*rhp23*Δ growth defects by Rpn10ΔUIM. Therefore, we considered other candidates that could have yet uncharacterized substrate recognition capabilities. Such candidates should either be proteasome subunits or proteasome-associated proteins and would be expected to display synthetic phenotypes with mutants in *rpn10* or *rhp23*. When searching the *Saccharomyces* Genome Database, we found that the proteasome subunit, called Sem1 in budding yeast (Funakoshi et al., 2004, Sone et al., 2004) and Dss1 in humans and fission yeast (Jossé et al., 2006), fulfills these criteria.

### Dss1 Is a Ubiquitin Binding Protein

**Figure 2:**
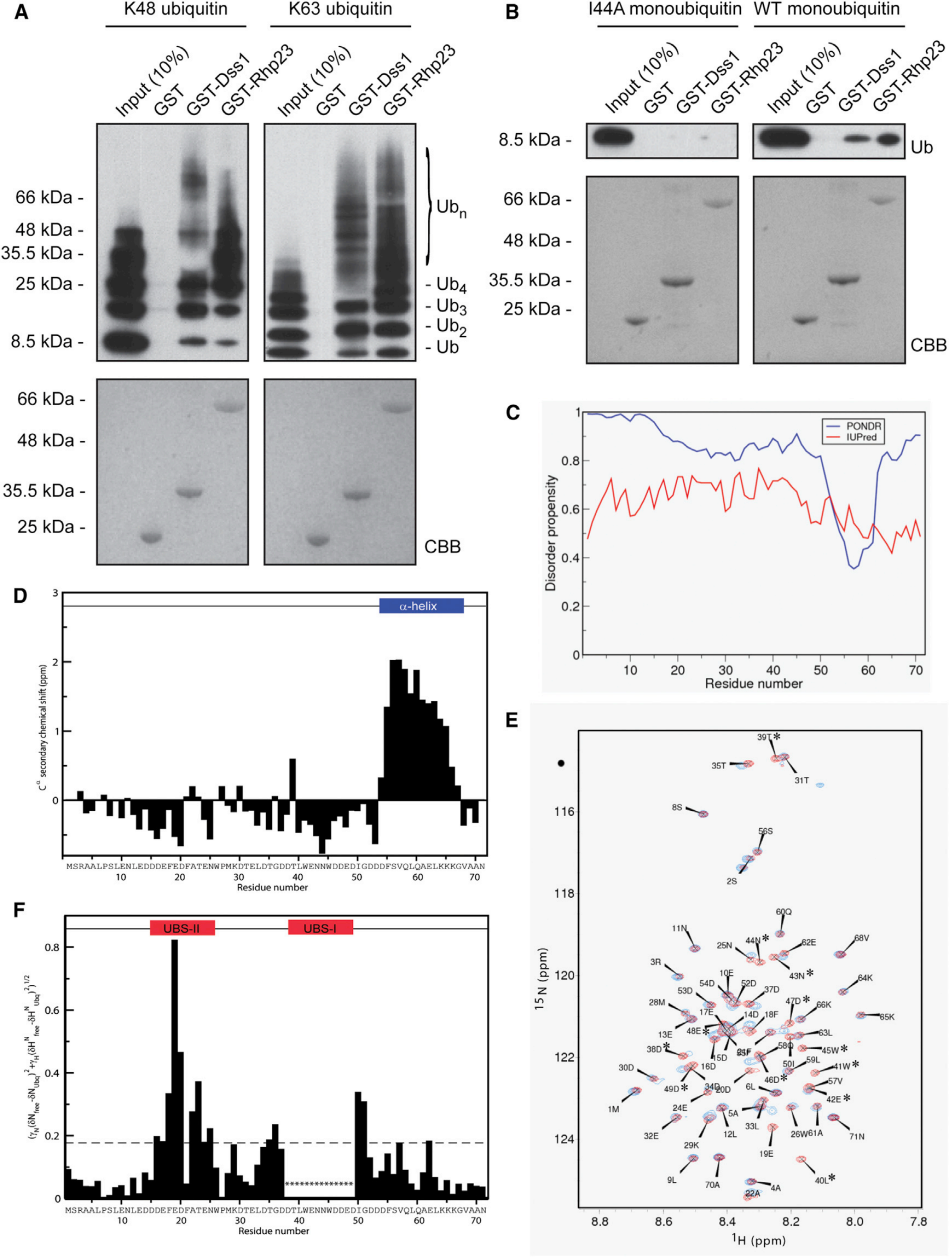
Dss1 Interacts Directly with Ubiquitin (A) K48- and K63-linked ubiquitin chains (3 μg per assay) (input) were coprecipitated with GST-Dss1. GST and GST-Rhp23 proteins were included as negative and positive controls, respectively. The precipitated material was analyzed by SDS-PAGE and western blotting using antibodies to ubiquitin. Equal loading was checked by staining with Coomassie brilliant blue (CBB). (B) I44A and wild-type (wt) monoubiquitin (10 μg) (input) were coprecipitated with GST-Dss1. GST and GST-Rhp23 proteins were included as negative and positive controls, respectively. The precipitated material was analyzed by SDS-PAGE and western blotting using antibodies to ubiquitin. Equal loading was checked by staining with CBB. (C) PONDR (blue) and IUPred (red) sequence analysis predicted Dss1 to be largely unstructured at physiological pH. PONDR predicted a short C-terminal stretch to be structured. (D) C^α^ secondary chemical shifts of Dss1 confirm the predominantly disordered structure. Positive C^α^ secondary chemical shifts identify α-helical structure in the C terminus from F55 through K66 indicated by a blue bar. (E) ^1^H-^15^N HSQC spectrum of Dss1 in the absence (red) and presence (blue) of a 50-fold molar excess of ubiquitin. Residues marked with an asterisk disappeared from the HSQC spectrum on addition of ubiquitin. (F) Plot of the per-residue calculated chemical shift perturbation (CSP) (see Supplemental Information) comparing identical samples of Dss1 in the absence and presence of a 50-fold molar excess of ubiquitin, revealing two UBSs, UBS-I and UBS-II, indicated by red bars. The horizontal dashed lines illustrate the average CSP and the average CSP plus 1 SD. Residues marked with an asterisk disappeared from the HSQC spectrum on addition of ubiquitin. See also Figure S2.

To assess if Dss1 functions as a proteasomal ubiquitin receptor, we first tested its ability to interact directly with ubiquitin chains. We performed an in vitro ubiquitin-binding assay using glutathione S-transferase (GST)-Dss1 and K48- and K63-linked ubiquitin chains. GST-Rhp23 was included as a positive control. Indeed, under these conditions, GST-Dss1 efficiently interacted with both K48 and K63 ubiquitin chains, while GST alone did not (Figure 2A).

In general, ubiquitin receptors recognize ubiquitin via a conserved hydrophobic patch around Ile44 (Husnjak et al., 2008). To test if Dss1 also binds ubiquitin via this hydrophobic area, we assayed the ability of Dss1 to interact with the I44A ubiquitin mutant. Compared to wild-type ubiquitin that clearly interacted with Dss1, I44A ubiquitin did not efficiently associate with Dss1 or Rhp23 (Figure 2B). This suggests that the ubiquitin Ile44 patch is important for efficient Dss1 and Rhp23 binding.

Scrutinizing the Dss1 sequence left us unable to identify any resemblance to known ubiquitin-binding sites (UBSs) or domains (Husnjak and Dikic, 2012). Structural prediction analyses of Dss1 suggested it to belong to the intrinsically disordered proteins (IDPs) (Figure 2C) (Uversky, 2011). PONDR (Obradovic et al., 2003), but not IUPred (Dosztányi et al., 2005), predicted that a short stretch in the Dss1 C terminus is structured (Figure 2C). To probe this further, we analyzed Dss1 by heteronuclear nuclear magnetic resonance (NMR) spectroscopy. Assigned C^α^ chemical shifts relative to random coil shifts (Figure 2D) (Kjaergaard et al., 2011), combined with a low-dispersion ^15^N,^1^H-heteronuclear single quantum correlation (HSQC) spectrum (Figure 2E; Figure S2A), conclusively identified Dss1 as intrinsically disordered with a single, transiently populated α helix from F55 through K66. Successive addition of excess ubiquitin and analysis by NMR uncovered two distinct UBSs, identified from chemical shift perturbation analyses. Titration analyses with increasing amounts of ubiquitin disclosed the strongest binding to ubiquitin by binding site I (UBS-I), which is located at D38–D49 (dissociation constant, K_D_, = 50 ± 30 μM) and disclosed the second and weakest site, UBS-II, located at D16–N25 (apparent K_D_ > 1 mM) (Figure 2F; Figures S2B and S2C). These UBSs are conserved and located in the disordered region of Dss1 (Figure S3). Notably, both sites have a similar sequence, characterized by a series of hydrophobic residues flanked by acidic residues (Figure S3).

### Dss1 Binds a Hydrophobic and Positively Charged Area on Ubiquitin

**Figure 3:**
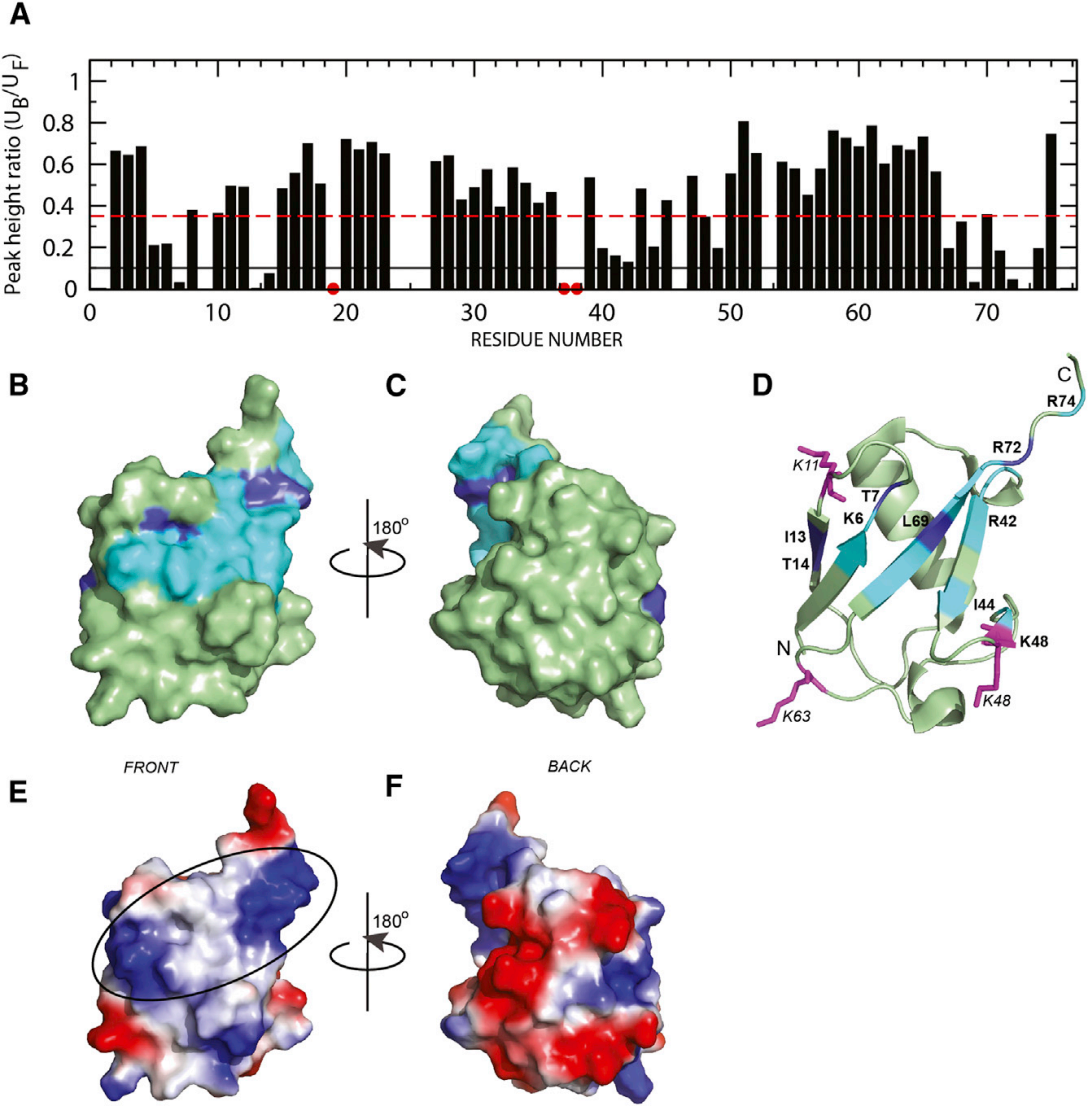
Dss1 Exploits a Tripartite Binding Site on Ubiquitin (A) Changes in peak intensities of ubiquitin in response to Dss1 binding. The red dashed line marks residues where the intensity decreased to less than 35%, and the black solid line marks those residues where the intensities are less than 10% of the unbound. The red dots mark proline residues not visible in the spectra. (B and C) Changes in peak intensities of ubiquitin by Dss1 addition mapped onto the 3D structure of ubiquitin (Protein Data Bank ID 1D3Z) (Cornilescu et al., 1998). The protein structure is shown in green. Light blue indicate residues with peak intensities decreased to less than 35%, and dark blue decreased to less than 10%. (B) is oriented as in (D) with the β sheet facing the viewer, and in (C), the opposite side is shown with the α helix facing the viewer. (D) Ribbon representation of ubiquitin with the same color coding as in (B) and with specific residues labeled. Three lysine residues, K11, K48, and K63 of ubiquitin are shown in magenta sticks. (E and F) Electrostatic surface representation of ubiquitin, calculated using PyMOL. Negative potentials are shown in red, positive potentials are shown in blue, and uncharged regions are shown in white. The tripartite Dss1 binding area is circled. (E) has the same orientation as in (B), and (F) has the same as in (C). See also Figure S3.

We subsequently mapped the corresponding interaction surface on ubiquitin by NMR, using ^13^C,^15^N-labeled ubiquitin (Figure 3). The perturbations of peak intensities of ubiquitin, imposed by addition of Dss1 (Figure 3A), mapped consistently to the surface-exposed common hydrophobic binding surface of ubiquitin involving the β sheet and the hydrophobic residues I13, L69, and I44 (Figures 3B–3D) but is also extended to the C terminus, resembling the binding site exploited by the E2 ubiquitin-conjugating enzyme Cdc34 (Arrigoni et al., 2012, Choi et al., 2010, Spratt and Shaw, 2011). Several positively charged residues located on the same surface were also significantly perturbed, whereas no perturbations were seen on the opposite face of ubiquitin (Figure 3C). A representation of the electrostatic surface of ubiquitin revealed a tripartite binding site of a hydrophobic patch flanked by two positively charged regions (Figures 3E and 3F). This directly mirrors the architecture of the UBSs identified in Dss1 (Figure S3). Moreover, the size of the interaction surface and the length of each UBS in Dss1 strongly suggest that the two UBSs bind independently to each their ubiquitin moiety. Of note, we observe that, depending on the linkages, there are unequal distances from the Dss1 binding site on ubiquitin to a second Dss1 binding site on a linked ubiquitin, suggesting that Dss1 may express a preference in the selection of different lysine-linked ubiquitin chains.

### Ubiquitin Binding Is Important for Dss1 Function

**Figure 4:**
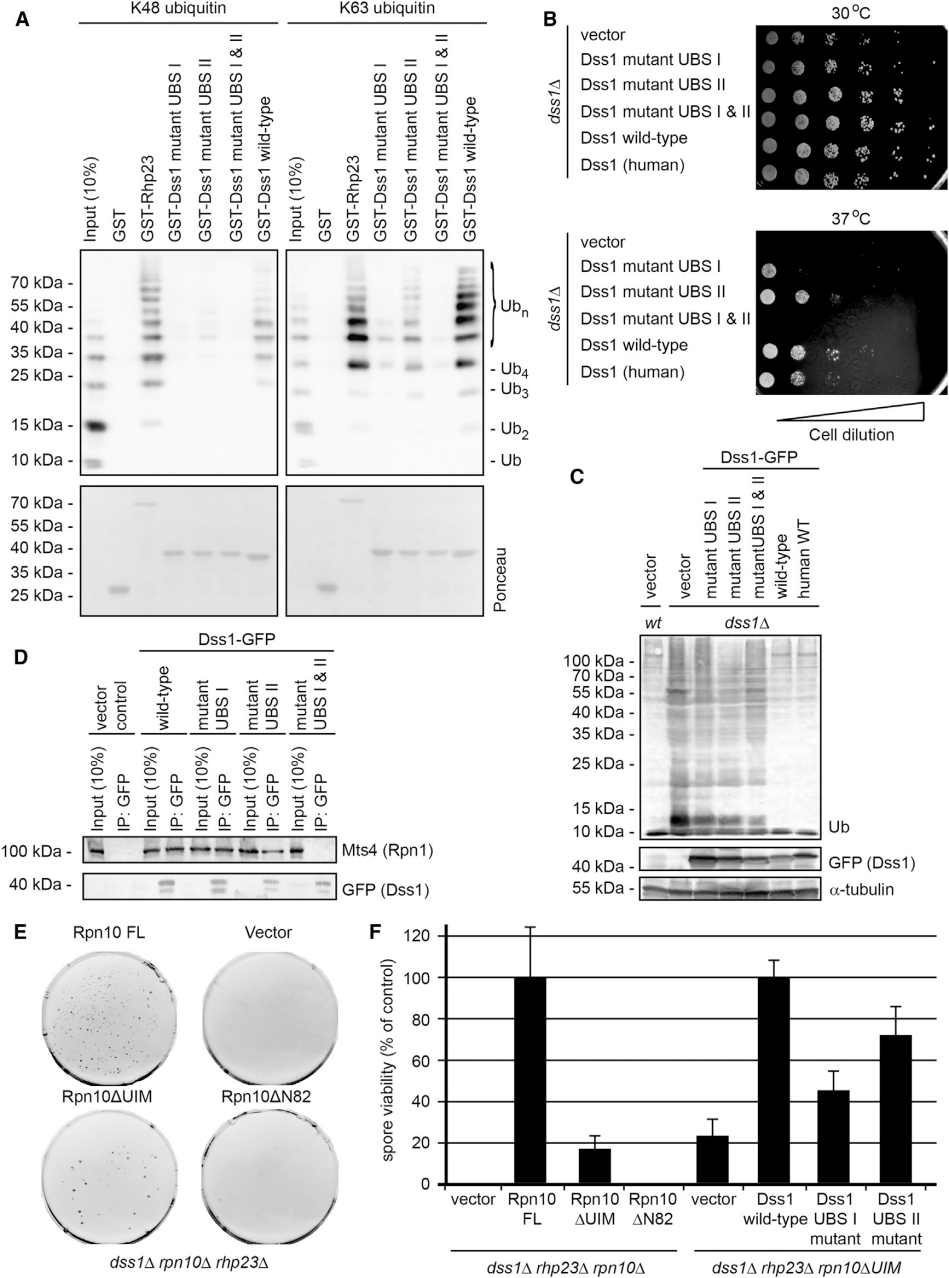
UBSs in Dss1 Are Required for Proteasome Function (A) K48-linked (left panel) and K63-linked (right panel) ubiquitin chains (input) (3 μg per assay) were coprecipitated with GST-Dss1, GST-Dss1 UBS-I mutant (L40A/W41A/W45A), GST-Dss1 UBS-II mutant (F18A/F21A/W26A), and GST-Dss1 UBS-I and UBS-II mutant (F18A/F21A/W26A/L40A/W41A/W45A). GST and GST-Rhp23 proteins were included as negative and positive controls, respectively. The precipitated material was analyzed by SDS-PAGE and western blotting using antibodies to ubiquitin. Equal loading was checked by staining with Ponceau S. (B) The *dss1*Δ strains transformed with the indicated expression constructs were analyzed for growth on solid media at 30°C and 37°C. The pictures were taken after 72 hr. (C) The *dss1*Δ strains transformed with the indicated expression constructs were analyzed for the presence of ubiquitin-protein conjugates by blotting. Expression of the various Dss1 proteins was confirmed by blotting for the GFP tag. Tubulin served as a loading control. wt, wild-type. (D) A *dss1*Δ strain was transformed with the indicated expression vectors for Dss1-GFP fusion proteins and used for immunoprecipitations with antibodies to GFP. The precipitated material was analyzed by SDS-PAGE and western blotting using antibodies to the proteasome subunit Mts4/Rpn1 and GFP on Dss1. Dss1 expression was not visible in whole cell lysates but was clearly enriched in the precipitated material. FL, full-length. (E) Plating assay of the *dss1*Δ*rhp23*Δ*rpn10*Δ strain with the indicated expression constructs. The *dss1*Δ*rpn10*Δ strain transformed with the indicated constructs was crossed to *dss1*Δ*rhp23*Δ cells to generate a triple deletion. Following crossing, 10,000 spores were plated under selection for the deleted genes and the expression vector. (F) Plating assays of the *dss1*Δ*rhp23*Δ*rpn10*Δ and *dss1*Δ*rhp23*Δ*rpn10*ΔUIM strains with the indicated Rpn10 and Dss1 expression constructs, as shown in Figure 4E were quantified. Following crossing, 10,000 spores were plated under selection for the deleted genes and the expression vectors. Viable spores were counted and normalized to the controls (Rpn10 FL and Dss1 wild-type). Data are presented as mean ± SEM (n = 6). See also Figure S4.

As expected from the NMR data, mutation of either UBS-I (L40A, W41A, W45A) or UBS-II (F18A, F21A, W26A) clearly reduced binding to ubiquitin, and no ubiquitin binding was observed for Dss1 mutated at both sites (Figure 4A). Consistent with UBS-I being the stronger of the two binding sites, mutation of this site also had a greater effect on ubiquitin binding (Figure 4A).

For better understanding of the functional relevance of Dss1 and the importance of its ubiquitin-binding activity, a range of yeast mutants was created and tested in growth assays under various conditions. Expression of Dss1 or any of the Dss1 variants did not affect cell growth of wild-type cells (Figure S4A), whereas deletion of the *dss1*^+^ gene resulted in a growth defect that was especially pronounced at higher temperatures (Figure 4B). When introducing the Dss1 variants into the *dss1*Δ strain, we observed that cells expressing Dss1, mutated at both UBS-I and UBS-II, displayed a significant growth defect (Figure 4B), while each of the single UBS mutants or wild-type human Dss1 only partially restored growth (Figure 4B). Similar effects were observed on media containing canavanine (Figure S4B), a drug that inhibits protein folding and induces cell stress. Notably, these genetic effects correlated with the cellular accumulation of ubiquitin-protein conjugates. Thus, ubiquitin-protein conjugates accumulated in the *dss1*Δ strain, and this accumulation was not affected by ectopic expression of Dss1 mutated in both UBS-I and UBS-II (Figure 4C). Expression of either Dss1 UBS-I or Dss1 UBS-II mutants partially reduced the level of ubiquitin conjugates in the *dss1*Δ strain, while expression of wild-type *S. pombe* Dss1 or human Dss1 fully reduced ubiquitin-protein conjugates to wild-type levels (Figure 4C).

We next analyzed if any of the Dss1 mutants were also compromised in proteasome binding. We found that wild-type Dss1, as well as individual Dss1 UBS-I and Dss1 UBS-II mutants, all efficiently coprecipitated 26S proteasomes (Figure 4D). However, Dss1 mutated in both UBS-I and UBS-II failed to interact with 26S proteasomes (Figure 4D). Hence, the strong phenotype of Dss1 mutated in both UBS-I and UBS-II is likely caused by both loss of ubiquitin binding and loss of proteasome binding. In contrast, the intermediate phenotypes of Dss1 with single mutations in UBS-II or, in particular, in UBS-I can likely be attributed to a reduced ubiquitin binding since they still bind to the proteasome.

Recently, Dss1 was shown to function in proteasome assembly (Tomko and Hochstrasser, 2014). To assess the importance of Dss1 on overall proteasome integrity, we isolated 26S proteasomes from a *dss1*Δ strain and analyzed them biochemically. We found that proteasomes lacking Dss1 still efficiently interacted with polyubiquitylated proteins (Figure S4C) and were proteolytically active (Figure S4D). This suggests that, structurally, 26S proteasomes are not strongly affected by loss of Dss1 and that the contribution of Dss1 to the proteasomal substrate binding capacity in vitro is lower compared to the already known substrate receptors. This agrees with previous in vitro activity studies of purified proteasomes, lacking all known UBSs, which suggest the existence of an additional low-affinity substrate binding site (Peth et al., 2010). To further rule out that the observed phenotype of the *dss1* null mutant was not caused by a general loss of 26S proteasome integrity, we performed label-free quantitative mass spectroscopy, comparing 26S proteasomes purified from wild-type, *rpn10*Δ, *rpn10*ΔUIM, and *dss1*Δ cells (Figures S4E and S4F). In agreement with data from budding yeast (Bohn et al., 2013, Tomko and Hochstrasser, 2014), loss of Dss1 caused a modest reduction in 26S proteasome integrity (Figures S4E–S4G). Mutation of the Dss1 UBS-I only slightly reduced the amount of Rpn10 in the 26S proteasome (Figure S4H). Loss of Rpn10 was more disruptive, with the amounts of 26S proteasomes being reduced to around 10% of that found in wild-type cells (Figures S4E–S4G).

Collectively, these data imply that ubiquitin binding is important for the function of Dss1 in the 26S proteasome in vivo and that Dss1 could be responsible for the viability of the *rhp23*Δ*rpn10*ΔUIM strain (Figure 1C). This being the case, then loss of Dss1 should impart growth defects in the *rhp23*Δ*rpn10*ΔUIM strain. Indeed, spore viability of the *dss1*Δ*rhp23*Δ*rpn10*ΔUIM strain was reduced compared to cells expressing the full-length Rpn10 protein (Figures 4E and 4F). When introducing wild-type Dss1 and the Dss1 UBS-I and UBS-II mutants in the *dss1*Δ*rhp23*Δ*rpn10*ΔUIM strain, we found that neither the Dss1 UBS-I mutant nor the Dss1 UBS-II mutant was able to fully restore growth of the *dss1*Δ*rhp23*Δ*rpn10*ΔUIM strain (Figure 4F), suggesting that the ubiquitin-binding function of Dss1, described here, is important for proteasomal function and cell viability.

## Discussion

In this article, we demonstrate that Dss1 has a previously uncharactized function as a ubiquitin-binding protein of the 26S proteasome: unlike other receptors, Dss1 interacts with ubiquitin via an unstructured UBS. Given the highly conserved nature of the UPS and the *dss1*^*+*^ gene itself (47% identity between fission yeast and human Dss1), and given that human Dss1 complements the phenotype of a fission yeast *dss1*Δ mutant, we propose that Dss1 acts as a ubiquitin receptor in all eukaryotes.

Most ubiquitin-binding proteins have well-defined and structured ubiquitin-binding domains or small motifs (Husnjak and Dikic, 2012). This is in sharp contrast to proteins interacting with the ubiquitin-like modifier SUMO that, in general, associate via short motifs located in intrinsically disordered regions (Vogt and Hofmann, 2012). The UBSs described here are both located in the disordered region of Dss1. We suspect that other ubiquitin-binding proteins may interact by a similar mechanism. In general, disordered proteins are not well conserved in sequence (Uversky, 2011), and by homology searches, we have not been able to identify other proteins containing any Dss1-like UBSs. However, we did note some similarity between the sites in Dss1 and the UBSs found in the E2-3R family of E2 ubiquitin-conjugating enzymes (Arrigoni et al., 2012) such as Cdc34 (Choi et al., 2010). Intriguingly, a recently described disordered region of Cdc34 binds an area on ubiquitin similar to the area we identified for Dss1 (Arrigoni et al., 2012, Choi et al., 2010, Spratt and Shaw, 2011), suggesting that these binding regions are required to be unstructured.

Previous studies in budding yeast have shown that cells lacking all known proteasomal UBSs still remain viable (Husnjak et al., 2008). The data presented here reveal that the same is true for fission yeast, but this viability, at least in part, depends on Dss1. What happens to ubiquitylated substrates after reaching the 26S proteasome, but prior to or during degradation, is still an open question. For instance, we know little about the events taking place during the initial substrate capture by Rpn10 and Rpn13, localized at the tip of the regulatory particle, and the translocation to the central ATPase ring. It is possible that substrates are handed over from the outer receptors to an inner receptor more proximal to the ATPase ring. The localization of Dss1 near the ATPase pore and the deubiquitylating subunit Rpn11 (Bohn et al., 2013) would fit such a model. The disordered and flexible nature of Dss1 could then allow for interaction with substrates presented in various orientations. However, like most disordered proteins (Uversky, 2011), Dss1 is multifunctional, even within the 26S proteasome, where it appears to act both structurally and functionally. This complicates the interpretation of the *dss1*Δ phenotypes. Recently, budding yeast Sem1 was shown to play an important role in proteasome assembly (Tomko and Hochstrasser, 2014). Specifically, Sem1 catalyzes incorporation of subunits Rpn3 and Rpn7 into the 19S regulatory complex through sites that overlap with UBS-I and UBS-II in fission yeast Dss1. However, this function of Sem1 becomes dispensable at later stages of proteasome assembly. Although our proteomic analyses of *dss1*Δ 26S proteasomes do not indicate that the level of Rpn3 or Rpn7 is reduced compared to that of other subunits of the lid complex, we also noted that Dss1, mutated in both UBS-I and UBS-II, is not incorporated into 26S proteasomes. Notably, the Dss1 mutant in UBS-I alone was still incorporated into 26S proteasomes but continued to display the temperature-dependent growth defect and ubiquitin-conjugate stabilization. This suggests that the phenotypes connected with the Dss1 ubiquitin-binding activity is limited to that of the Dss1 UBS-I, which has a much greater affinity for ubiquitin compared to UBS-II. However, Dss1 also has proteasome-independent functions, including associating with DNA repair proteins (Yang et al., 2002) and the transcription-export complex (Ellisdon et al., 2012, Faza et al., 2009). We speculate that the ubiquitin-binding activity of Dss1 may also play a functional role for these cellular processes.

In conclusion, our studies suggest the intrinsically disordered protein Dss1 as a ubiquitin receptor for the 26S proteasome in fission yeast. Since Dss1 is phylogenetically conserved, we propose that Dss1 acts as a ubiquitin receptor in all eukaryotes.

## Experimental Procedures

### Yeast Strains and Protocols

All strains used for this work are listed in Table S1. The strains were all derived from the *S. pombe* wild-type heterothallic 972h^−^ and 975h^+^. Standard genetic methods and media were used (Moreno et al., 1991).

### Fission Yeast Expression Plasmids

The plasmids used for expression of *rpn10*^*+*^ and *dss1*^*+*^ in fission yeast were pREP41 carrying the budding yeast *LEU2* gene for selection and the *nmt41* promoter or the pDUAL vector carrying *ura4*^+^ for selection and the *nmt1* promoter (Matsuyama et al., 2004).

### Antibodies

Antibodies to Mts4/Rpn1 have been described elsewhere (Wilkinson et al., 2001). Other antibodies were commercially available: flag (Sigma), green fluorescent protein (GFP; Sigma), tubulin (Abcam), 20S proteasome MCP231 (Enzo), T7 (Bethyl), and ubiquitin (DAKO).

### Protein Purification and Coprecipitation Assays

The 26S proteasomes, flag-tagged on Mts4 (Rpn1), were purified as described elsewhere (Verma et al., 2002).

### Proteasome Assays

The proteolytic activity of affinity-purified 26S proteasomes with or without Dss1 was measured in the presence or absence of 5 μM of the proteasome inhibitor Bortezomib (LC Laboratories) using the suc-LLVY-AMC substrate (Enzo) as described elsewhere (Groll et al., 2006).

### Mass Spectrometry

Detailed methods are provided in the Supplemental Information.

### Purification of Recombinant Proteins and Coprecipitation Assays

All Dss1 proteins were expressed in *Escherichia coli* BL21 (DE3) from the pGEX6P1 or pDEST15 vectors by standard methods. Harvested cells were lysed by sonication in a buffer containing 12.5 mM Tris-HCl, pH 7.5, 37.5 mM NaCl, 1 mM phenylmethylsulfonyl fluoride and cOmplete Mini Protease Inhibitor Tablets (Roche). Following centrifugation at 13,000 × *g*, the cleared lysates were tumbled with glutathione-sepharose beads (GE Healthcare) for 1 hr at 4°C and extensively washed with the lysis buffer. Coprecipitation assays were performed as described elsewhere (Wilkinson et al., 2001). For the ubiquitin precipitation studies, 3 μg of K48- and K63-linked ubiquitin chains (Boston Biochemicals) were used per precipitation in 100 μl buffer A, containing 12.5 mM Tris-HCl, pH 7.5, 37.5 mM NaCl. The protein/bead ratio was adjusted to about 1 mg/ml, and 10 μl of beads were used per assay. After 2 hr of tumbling at 4°C, the beads were washed twice with 1 ml of buffer A with 0.5% Triton X-100 and once with buffer A. Bound protein was eluted by boiling with SDS sample buffer. Some ubiquitin blots were boiled for 30 min after transfer to enhance reactivity and blocked with 5% BSA in PBS.

The T7-tagged Sic1-PY was purified and in vitro ubiquitylated as described elsewhere (Kriegenburg et al., 2008).

### NMR Samples and Recordings

Detailed methods are provided in the Supplemental Information.

## Author Contributions

K.P., F.K., B.M., and I.B.L. performed the cloning and complementation studies. K.P., F.K., B.M., and C.G. performed the genetics. K.P. performed the protein purification experiments in Figures 2A, 2B, and S4. F.K. and I.B.L. performed the coprecipitation experiments in Figure 4. H.R., R.B., and B.B.K. performed the protein purification and NMR studies and analyses. M.H.T. performed proteomic analyses and edited the manuscript. K.P., F.K., B.M., K.G.H., B.B.K., R.H.-P., and C.G. designed the study. K.P., R.H.-P., R.T.H., B.B.K., and C.G. analyzed the data. K.P., B.B.K., R.H.-P., and C.G. wrote the paper.

## Supplemental Information

Document S1. Figure S1–S4, Table S1, and Supplemental Experimental Procedures

Document S2. Article plus Supplemental Information

## References

[bib1] Arrigoni A., Grillo B., Vitriolo A., De Gioia L., Papaleo E. (2012). C-Terminal acidic domain of ubiquitin-conjugating enzymes: a multi-functional conserved intrinsically disordered domain in family 3 of E2 enzymes. J. Struct. Biol..

[bib2] Bohn S., Sakata E., Beck F., Pathare G.R., Schnitger J., Nágy I., Baumeister W., Förster F. (2013). Localization of the regulatory particle subunit Sem1 in the 26S proteasome. Biochem. Biophys. Res. Commun..

[bib3] Choi Y.S., Wu K., Jeong K., Lee D., Jeon Y.H., Choi B.S., Pan Z.Q., Ryu K.S., Cheong C. (2010). The human Cdc34 carboxyl terminus contains a non-covalent ubiquitin binding activity that contributes to SCF-dependent ubiquitination. J. Biol. Chem..

[bib4] Cornilescu G., Marquardt J.L., Ottiger M., Bax A. (1998). Validation of protein structure from anisotropic carbonyl chemical shifts in a dilute liquid crystalline phase. J. Am. Chem. Soc..

[bib5] Deveraux Q., Ustrell V., Pickart C., Rechsteiner M. (1994). A 26 S protease subunit that binds ubiquitin conjugates. J. Biol. Chem..

[bib6] Dosztányi Z., Csizmok V., Tompa P., Simon I. (2005). IUPred: web server for the prediction of intrinsically unstructured regions of proteins based on estimated energy content. Bioinformatics.

[bib7] Ellisdon A.M., Dimitrova L., Hurt E., Stewart M. (2012). Structural basis for the assembly and nucleic acid binding of the TREX-2 transcription-export complex. Nat. Struct. Mol. Biol..

[bib8] Faza M.B., Kemmler S., Jimeno S., González-Aguilera C., Aguilera A., Hurt E., Panse V.G. (2009). Sem1 is a functional component of the nuclear pore complex-associated messenger RNA export machinery. J. Cell Biol..

[bib9] Finley D. (2009). Recognition and processing of ubiquitin-protein conjugates by the proteasome. Annu. Rev. Biochem..

[bib10] Funakoshi M., Li X., Velichutina I., Hochstrasser M., Kobayashi H. (2004). Sem1, the yeast ortholog of a human BRCA2-binding protein, is a component of the proteasome regulatory particle that enhances proteasome stability. J. Cell Sci..

[bib11] Groll M., Berkers C.R., Ploegh H.L., Ovaa H. (2006). Crystal structure of the boronic acid-based proteasome inhibitor bortezomib in complex with the yeast 20S proteasome. Structure.

[bib12] Hartmann-Petersen R., Seeger M., Gordon C. (2003). Transferring substrates to the 26S proteasome. Trends Biochem. Sci..

[bib13] Husnjak K., Dikic I. (2012). Ubiquitin-binding proteins: decoders of ubiquitin-mediated cellular functions. Annu. Rev. Biochem..

[bib14] Husnjak K., Elsasser S., Zhang N., Chen X., Randles L., Shi Y., Hofmann K., Walters K.J., Finley D., Dikic I. (2008). Proteasome subunit Rpn13 is a novel ubiquitin receptor. Nature.

[bib15] Jossé L., Harley M.E., Pires I.M., Hughes D.A. (2006). Fission yeast Dss1 associates with the proteasome and is required for efficient ubiquitin-dependent proteolysis. Biochem. J..

[bib16] Kjaergaard M., Brander S., Poulsen F.M. (2011). Random coil chemical shift for intrinsically disordered proteins: effects of temperature and pH. J. Biomol. NMR.

[bib17] Kriegenburg F., Seeger M., Saeki Y., Tanaka K., Lauridsen A.M., Hartmann-Petersen R., Hendil K.B. (2008). Mammalian 26S proteasomes remain intact during protein degradation. Cell.

[bib18] Matsuyama A., Shirai A., Yashiroda Y., Kamata A., Horinouchi S., Yoshida M. (2004). pDUAL, a multipurpose, multicopy vector capable of chromosomal integration in fission yeast. Yeast.

[bib19] Mayor T., Graumann J., Bryan J., MacCoss M.J., Deshaies R.J. (2007). Quantitative profiling of ubiquitylated proteins reveals proteasome substrates and the substrate repertoire influenced by the Rpn10 receptor pathway. Mol. Cell. Proteomics.

[bib20] Moreno S., Klar A., Nurse P. (1991). Molecular genetic analysis of fission yeast Schizosaccharomyces pombe. Methods Enzymol..

[bib21] Obradovic Z., Peng K., Vucetic S., Radivojac P., Brown C.J., Dunker A.K. (2003). Predicting intrinsic disorder from amino acid sequence. Proteins.

[bib22] Peth A., Uchiki T., Goldberg A.L. (2010). ATP-dependent steps in the binding of ubiquitin conjugates to the 26S proteasome that commit to degradation. Mol. Cell.

[bib23] Saeki Y., Tanaka K. (2008). Cell biology: two hands for degradation. Nature.

[bib24] Sakata E., Bohn S., Mihalache O., Kiss P., Beck F., Nagy I., Nickell S., Tanaka K., Saeki Y., Förster F., Baumeister W. (2012). Localization of the proteasomal ubiquitin receptors Rpn10 and Rpn13 by electron cryomicroscopy. Proc. Natl. Acad. Sci. USA.

[bib25] Schreiner P., Chen X., Husnjak K., Randles L., Zhang N., Elsasser S., Finley D., Dikic I., Walters K.J., Groll M. (2008). Ubiquitin docking at the proteasome through a novel pleckstrin-homology domain interaction. Nature.

[bib26] Seeger M., Hartmann-Petersen R., Wilkinson C.R., Wallace M., Samejima I., Taylor M.S., Gordon C. (2003). Interaction of the anaphase-promoting complex/cyclosome and proteasome protein complexes with multiubiquitin chain-binding proteins. J. Biol. Chem..

[bib27] Sone T., Saeki Y., Toh-e A., Yokosawa H. (2004). Sem1p is a novel subunit of the 26 S proteasome from Saccharomyces cerevisiae. J. Biol. Chem..

[bib28] Spratt D.E., Shaw G.S. (2011). Association of the disordered C-terminus of CDC34 with a catalytically bound ubiquitin. J. Mol. Biol..

[bib29] Su V., Lau A.F. (2009). Ubiquitin-like and ubiquitin-associated domain proteins: significance in proteasomal degradation. Cell. Mol. Life Sci..

[bib30] Tomko R.J., Hochstrasser M. (2014). The intrinsically disordered Sem1 protein functions as a molecular tether during proteasome lid biogenesis. Mol. Cell.

[bib31] Uversky V.N. (2011). Intrinsically disordered proteins from A to Z. Int. J. Biochem. Cell Biol..

[bib32] Verma R., Aravind L., Oania R., McDonald W.H., Yates J.R., Koonin E.V., Deshaies R.J. (2002). Role of Rpn11 metalloprotease in deubiquitination and degradation by the 26S proteasome. Science.

[bib33] Verma R., Oania R., Graumann J., Deshaies R.J. (2004). Multiubiquitin chain receptors define a layer of substrate selectivity in the ubiquitin-proteasome system. Cell.

[bib34] Vogt B., Hofmann K. (2012). Bioinformatical detection of recognition factors for ubiquitin and SUMO. Methods Mol. Biol..

[bib35] Wilkinson C.R., Seeger M., Hartmann-Petersen R., Stone M., Wallace M., Semple C., Gordon C. (2001). Proteins containing the UBA domain are able to bind to multi-ubiquitin chains. Nat. Cell Biol..

[bib36] Yang H., Jeffrey P.D., Miller J., Kinnucan E., Sun Y., Thoma N.H., Zheng N., Chen P.L., Lee W.H., Pavletich N.P. (2002). BRCA2 function in DNA binding and recombination from a BRCA2-DSS1-ssDNA structure. Science.

