# Supplementary material for: Dss1 Is a 26S Proteasome Ubiquitin Receptor
Source: Mol Cell. 2014 Nov 6;56(3):453–61. doi: 10.1016/j.molcel.2014.09.008 (PMC4232310; doi:10.1016/j.molcel.2014.09.008)
Supplement: Document S1. Figure S1–S4, Table S1, and Supplemental Experimental Procedures [file mmc1.pdf]

Molecular Cell, Volume *56*

Supplemental Information

Dss1 Is a 26S Proteasome Ubiquitin Receptor

Konstantinos Paraskevopoulos, Franziska Kriegenburg, Michael H. Tatham, Heike I. Rösner, Bethan Medina, Ida B. Larsen, Rikke Brandstrup, Kevin G. Hardwick, Ronald T. Hay, Birthe B. Kragelund, Rasmus Hartmann-Petersen, and Colin Gordon

Supplemental Figures

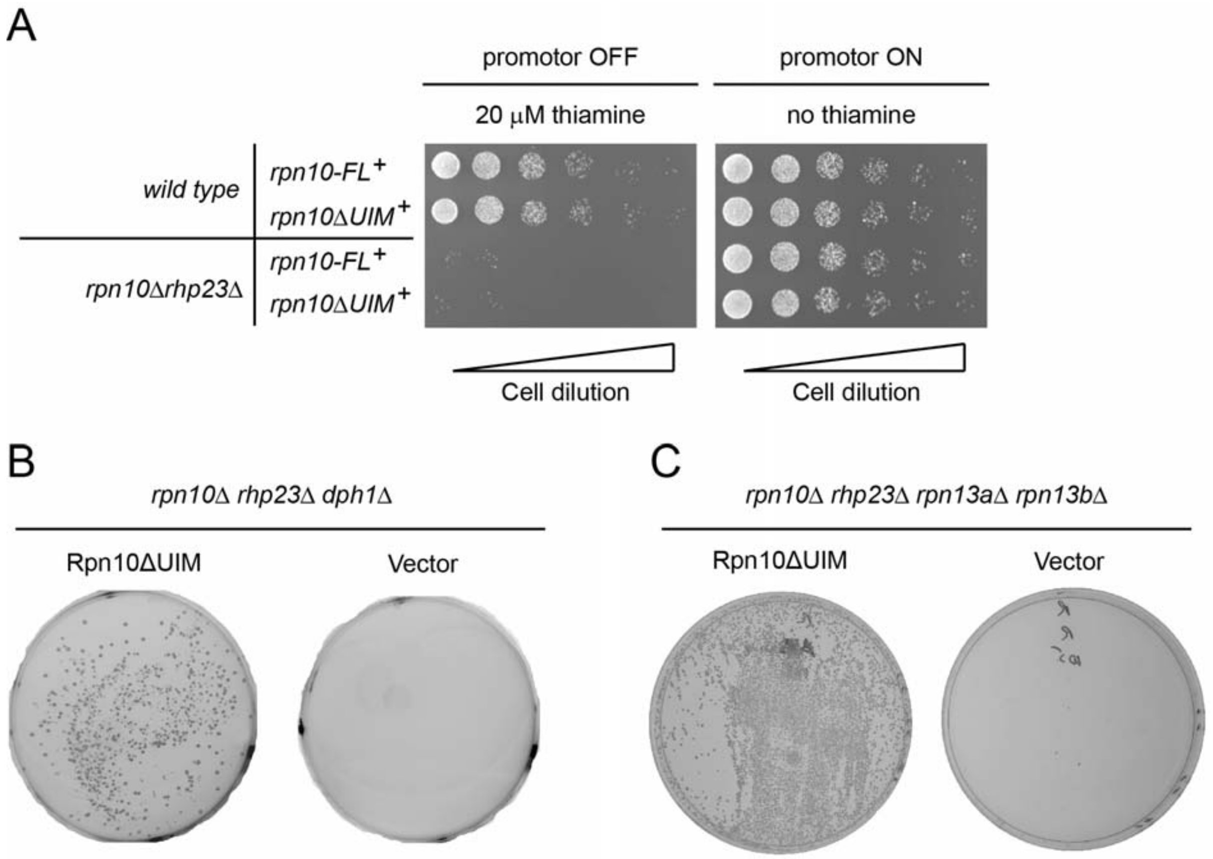

Figure S1 *Further complementation studies with Rpn10ΔUIM as in Figure 1.* (A) Wild type and *rpn10*Δ*rhp23*Δ strains, containing the *rpn10-FL* (full length) or *rpn10*Δ*UIM* thiamine-regulated expression constructs were compared in growth assays on media with thiamine (expression off) or without thiamine (expression on). (B) Rpn10ΔUIM and a control plasmid (vector) were stably integrated into *rpn10*Δ*dph1*Δ and *rhp23*Δ*dph1*Δ deletion strains. The two strains were crossed to each other to generate a triple deletion. Following crossing, 10,000 spores were plated on media that selected for the deletion mutants and the expression vector. (C) Rpn10ΔUIM and a control plasmid (vector) were stably integrated into *rpn10*Δ*rpn13a*Δ*rpn13b*Δ and *rhp23*Δ*rpn13a*Δ*rpn13b*Δ deletion strains. The two strains were crossed to each other to generate a quadruple deletion. Following crossing, 10,000 spores were plated on media that selected for the deletion mutants and the expression vector.

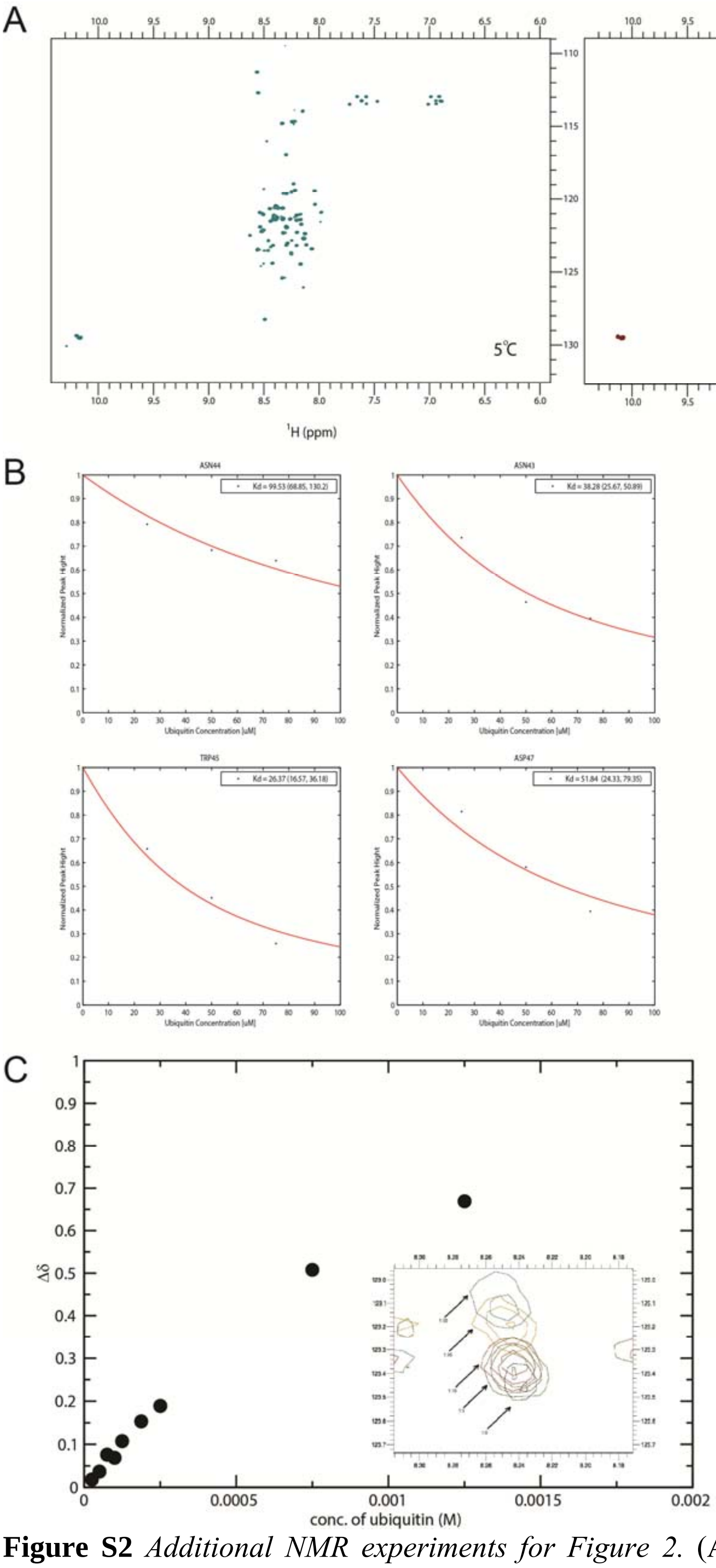

Figure S2 *Additional NMR experiments for Figure 2.* (A) ^{15}N,^{1}H-HSQC NMR spectra of Dss1 recorded at 5 °C (left panel, green) and 25 °C (right panel, red). (B) Titration experiments using ^{13}C,^{15}N-labelled DSS1 and unlabelled ubiquitin. Peak intensities for ubiquitin binding site I (UBS-I) are plotted as a function of increasing concentration of ubiquitin and fitted to a hyperbolic binding curve. (C) For ubiquitin binding site II (UBS-II),

no binding curve could be fitted, as the titration did not reach saturation. The insert shows the HSQC cross peak of residue 19E at increasing concentrations of ubiquitin.

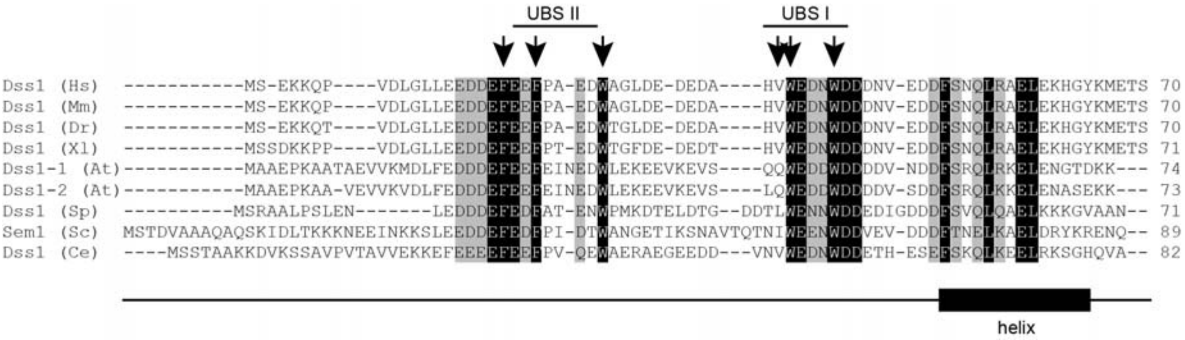

Figure S3 *Dss1 alignment showing the UBS regions that bind to the area in ubiquitin marked in Figure 3.* ClustalW alignments of human (Hs), mouse (Mm), fruit fly (Dm), frog (Xl), plant (At), fission yeast (Sp), budding yeast (Sc) and worm (Ce) Dss1. Conserved residues have been shaded. The helical area is shown by the bar. Ubiquitin binding site 1 (UBS-I) and ubiquitin binding site 2 (UBS-II) are conserved.

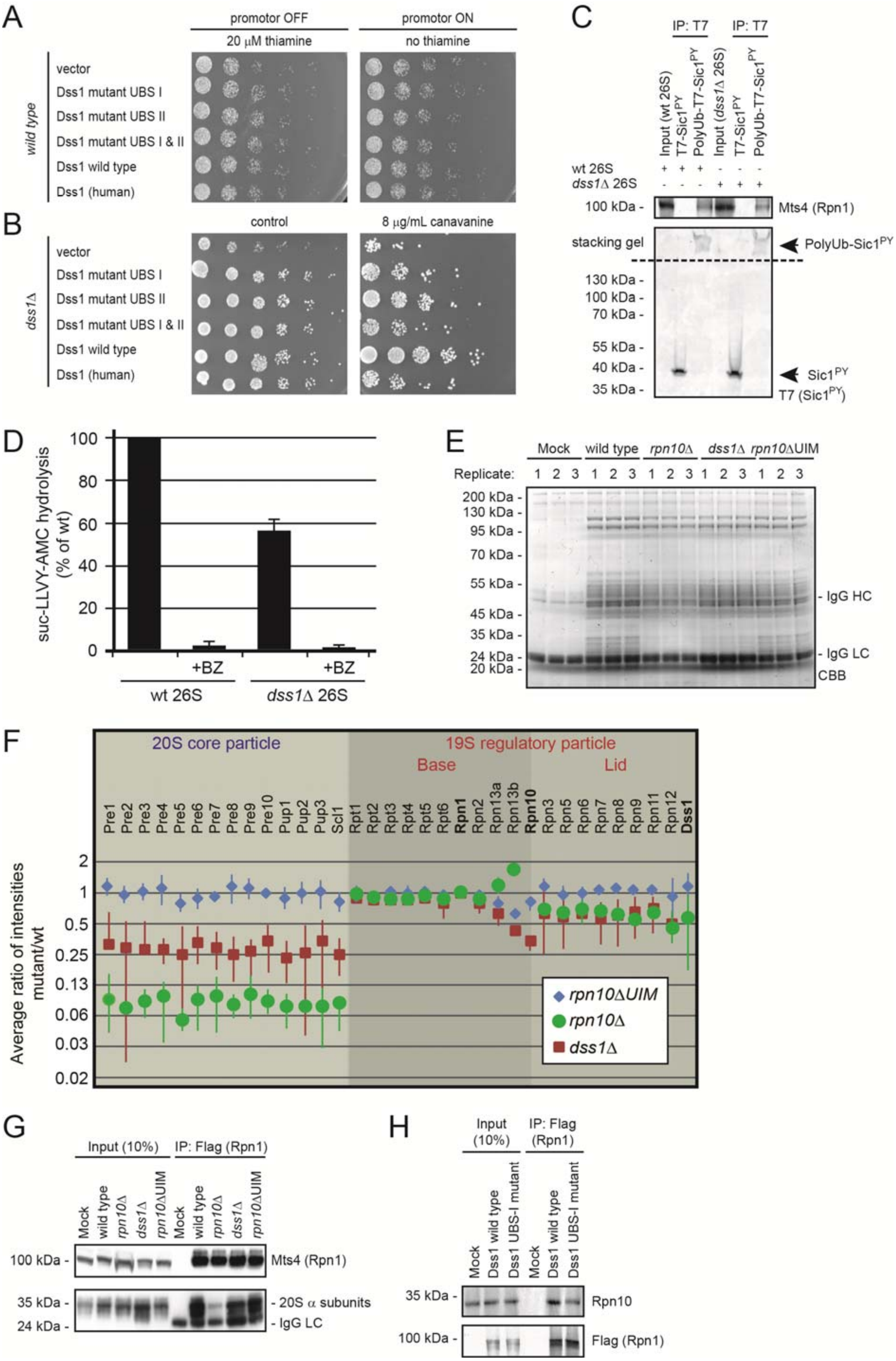

A
promotor OFF
20 μM thiamine
promotor ON
no thiamine
wild type
vector
Dss1 mutant UBS I
Dss1 mutant UBS II
Dss1 mutant UBS I & II
Dss1 wild type
Dss1 (human)
B
control
8 μg/mL canavanine
dss1Δ
C
IP: T7
IP: T7
Input (wt 26S)
T7-Sic1PY
PolyUb-T7-Sic1PY
Input (dss1Δ 26S)
wt 26S
dss1Δ 26S
100 kDa -
Mts4 (Rpn1)
stacking gel
PolyUb-Sic1PY
130 kDa -
70 kDa -
55 kDa -
40 kDa -
35 kDa -
Sic1PY
T7 (Sic1PY)
D
suc-LLVY-AMC hydrolysis (% of wt)
+BZ
wt 26S
dss1Δ 26S
E
Mock
wild type
rpn10Δ
dss1Δ
rpn10ΔUIM
Replicate:
200 kDa -
95 kDa -
45 kDa -
24 kDa -
20 kDa -
- IgG HC
- IgG LC
CBB
F
20S core particle
19S regulatory particle
Base
Lid
Pre1 Pre2 Pre3 Pre4 Pre5 Pre6 Pre7 Pre8 Pre9 Pre10 Pup1 Pup2 Pup3 Scl1 Rpt1 Rpt2 Rpt3 Rpt4 Rpt5 Rpt6 Rpn1 Rpn2 Rpn13a Rpn13b Rpn10 Rpn3 Rpn5 Rpn6 Rpn7 Rpn8 Rpn9 Rpn11 Rpn12 Dss1
Average ratio of intensities mutant/wt
2 1 0.5 0.25 0.13 0.06 0.03 0.02
rpn10ΔUIM
rpn10Δ
dss1Δ
G
Input (10%)
IP: Flag (Rpn1)
Mock
wild type
rpn10Δ
dss1Δ
rpn10ΔUIM
100 kDa -
Mts4 (Rpn1)
35 kDa -
24 kDa -
- 20S α subunits
- IgG LC
H
Input (10%)
IP: Flag (Rpn1)
Mock
Dss1 wild type
Dss1 UBS-I mutant
35 kDa -
Rpn10
100 kDa -
Flag (Rpn1)

Figure S4 *Control experiments for Figure 4*. (A) Wild type *S. pombe* cells, containing the indicated Dss1 thiamine-regulated expression constructs, were compared in growth assays on media with thiamine (expression off) or without thiamine (expression on). (B) The growth of *dss1*Δ strains, containing the indicated Dss1 expression constructs, was compared on media with (right panel) or without (left panel) canavanine. (C) T7-tagged Sic1-PY and Sic1-PY, which had been *in vitro* ubiquitylated were used in immunoprecipitation (IP) experiments with purified wild type 26S proteasome or 26S proteasomes, purified from a *dss1*Δ mutant. Note that the poly-ubiquitylated Sic1-PY does not migrate into the separation gel but stays in the stacking gel. The presence of 26S proteasomes and Sic1-PY was determined by blotting, using antibodies to the 19S regulatory complex subunit Mts4 (Rpn1) and the T7-tag on Sic1-PY. (D) Hydrolysis of suc-LLVY-AMC substrate, determined for affinity purified Rpn1-flag tagged 26S proteasome (wt 26S) and 26S proteasome without Dss1 (*dss1*Δ 26S) with and without the proteasome inhibitor Bortezomib (BZ). Error bars indicate the S.E.M. (n = 4). The activities were normalized to the amount of precipitated Rpn1. (E) The Rpn1-flag tagged 26S proteasome preparations from wild-type yeast, *dss1*Δ cells, *rpn10*Δ cells, and *rpn10*Δ*UIM* cells analyzed by SDS-PAGE. An untagged (Mock) strain was included as a negative control. The gel was stained with Coomassie Brilliant Blue (CBB). The IgG heavy chain (HC) and light chain (LC) from the anti-flag antibodies are marked. (F) Plot showing average ratios of the indicated protein intensity; mutant/wt cells. Protein members of the 20S core particle, 19S regulatory particle base and lid subcomplexes are shown. The intensities for each type were normalized to the intensity of Rpn1. (G) Rpn1-flag tagged 26S proteasomes were immunoprecipitated using antibodies to the flag epitope and analyzed by SDS-PAGE and blotting with antibodies to the 19S complex subunit Mts4 (Rpn1) or the 20S α subunits. The IgG light chain (LC) from the anti-flag antibody is marked. (H) Rpn1-flag tagged 26S proteasomes were immunoprecipitated from a wild type or *dss1* UBS-I mutant background using antibodies to the flag epitope and analyzed by SDS-PAGE and blotting with antibodies to Rpn10 and the flag epitope on Rpn1.

Supplemental Table

Table S1

Strains used in this study

Strain	Source
ura4-D18 leu1-32 ade6	Lab stock
rhp23::ura4$^+$ura4-D18 leu1.32	(Wilkinson et al., 2001)
rhp23::G418^R ura4-D18 leu1.32	This study
rpn10::Nat^R ura4-D18 leu1.32	This study
dph1::G418^R ura4-D18 leu1.32	This study
dss1::G418^R ura4-D18 leu1.32	This study
dss1:: ura4$^+$ura4-D18 leu1.32	(Mannen et al., 2008)
rpn13a::G418^R ura4-D18 leu1.32	This study
rpn13b::Ble^R ura4-D18 leu1.32	This study
mts4::mts4-Flag(G418^R) ura4-D18 leu1.32	This study
dph1::G418^R rhp23::ura4$^+$ ura4-D18 leu1.32	This study
dph1::G418^R rpn10::Nat^R ura4-D18 leu1.32	This study
rpn13a::G418^R rpn13b::Ble^R rhp23::ura4$^+$ ura4-D18 leu1.32	This study
rpn13a::G418^R rpn13b::Ble^R rpn10::Nat^R ura4-D18 leu1.32	This study
dss1::ura4$^+$ rhp23::G418^R ura4-D18 leu1.32	This study
dss1::ura4$^+$ rpn10::Nat^R ura4-D18 leu1.32	This study

Supplemental Methods

Mass spectrometry

About 20 µg of each batch of purified 26S proteasomes was fractionated twice on 12 % NuPAGE gels (Invitrogen). Two rounds of in-gel peptide preparation were made, first using GluC digestion and second using trypsin (Shevchenko et al., 2006). Peptides were alkylated with chloroacetamide. Peptide samples were analyzed by LC-MS/MS on a Q Exactive mass spectrometer (Thermo Scientific) coupled to an EASY-nLC 1000 liquid chromatography system via an EASY-Spray ion source (Thermo Scientific) running a 75 µm x 500 mm EASY-Spray column. Elution gradient durations of 60 minutes (GluC) and 150 minutes (trypsin) were used. Data were acquired in the data-dependent mode. Full scan spectra (*m/z* 304-1800) were acquired with resolution R = 70,000 at *m/z* 400 (after accumulation to a target value of 1,000,000 with maximum injection time of 20 ms). The 10 most intense ions were fragmented by HCD and measured with a target value of 500,000, maximum injection time of 60 ms and intensity threshold of $1.7e^3$. A 40 second dynamic exclusion list was applied.

Raw MS data files were processed together with the quantitative MS processing software MaxQuant (version 1.3.0.5) (Cox et al., 2011; Cox and Mann, 2008). Enzyme specificity was set to GluC or trypsin-P as required. Cysteine carbamidomethylation was selected as a fixed modification and methionine oxidation, protein N-acetylation and gly-gly adducts to lysine were chosen as variable modifications. The data were searched against a target/decoy *S. pombe* database. Initial maximum allowed mass deviation was set to 20 parts per million (ppm) for peptide masses and 0.5 Da for MS/MS peaks. The minimum peptide length was set

to seven amino acids and a maximum of four missed cleavages. 1 % false discovery rate (FDR) was required at both the protein and peptide level. In addition to the FDR threshold, proteins were considered identified if they had at least four unique peptides. The 'match between runs' option was selected with a time window of two minutes. Data were output such that each digestion of each gel slice was considered a single 'experiment', so protein intensity values based on extracted ion chromatograms were reported for each. After internal normalization across comparable samples, the intensities for each preparation were summed to provide a single intensity value for each protein in each proteasome preparation. Each protein intensity value was normalized to the total protein intensity of Rpn1. These intensities were used as an approximation of relative protein abundance for comparing the same protein among samples.

NMR samples and recordings

For the assignment, an 80 μM Dss1 solution was prepared in a 25 mM Tris/HCl pH 7.5, 50 mM NaCl, 10 % D_2O (v/v), 12.5 μM DSS (2,2-dimethyl-2-silanepentane-5-sulfonic acid), pH 7.5. The backbone resonances were assigned from the HNCA, HNCOCA, HNN and a ^{15}N-edited NOESY-HSQC, on a Varian INOVA 800 MHz spectrometer, using standard pulse programs from the Varian BioPack, with the following parameters: 2D ^{1}H^{15}N HSQC: 2048 complex points (t_2), 256 increments (t_1), spectral widths (SWs) = 13020.8 Hz (1H) and 2500 Hz (15N), nt = 16, recorded at 5 ºC and 25 ºC (Fig. S3B). 3D HNCA: 2048 complex points (t_3), 90 (t_2) and 20 (t_1), SWs = 13020.8 Hz (^{1}H), 2413.4 Hz (^{13}C) and 972.6 Hz (^{15}N), nt = 8. 3D HN(CO)CA: 2048 complex points (t_3), 90 (t_2) and 20 (t_1) increments in the t_2 and t_1 dimensions, SWs = 13020.8 2 Hz (^{1}H), 2413.4 Hz (^{13}C) and 972.6 Hz (^{15}N), nt = 8. 3D HNN: 2048 complex points (t_3), 40 (t_2) and 12 (t_1) increments, SWs = 13020.8 Hz (^{1}H) and 972.6 Hz (^{15}N), nt= 64. 3D ^{15}N-edited NOESY-HSQC: 2048 complex points (t_3), 100 (t_2) and 20

(t_1) increments, SWs = 13020.8 Hz (^{1}H) and 972.6 Hz (^{15}N), mixing time = 150 ms, nt = 32. All 3D experiments were recorded using non-linear sampling with a 25% data reduction according to the Orekhov method incorporated into the Varian BioPack.

For the ubiquitin binding study, two Dss1 stock solutions of 25 µM were prepared in a 50 mM phosphate buffer, 100 mM NaCl, 10 % D_2O (v/v), 12.5 µM DSS, pH 7.5. One stock solution also contained 250 µM unlabelled ubiquitin from bovine erythrocytes (Sigma-Aldrich). ^{15}N chemical shifts were obtained from ^{1}H^{15}N - HSQC spectra recorded at 5 ºC with 2048 complex points (t_2), 256 increments in the t_1 dimension, SWs = 13020.8 (^{1}H) and 1945.2 Hz (^{15}N), nt=24. Assignment of the bound state of Dss1 was aided by inclusion of triple-resonance HNCO, HNCA, and HNCACB spectra recorded analogously to those used for the initial assignments.

For mapping the Dss1 binding on ubiquitin, ^{15}N-edited HSQC spectra were recorded for two identical samples of 20 µM ^{15}N^{13}C labelled ubiquitin, prepared in a buffer containing 50 mM NaCl and 25 mM Tris/HCl pH 7.5. One sample also contained a 5-fold molar excess of unlabelled Dss1. Assignment for ubiquitin was taken from the literature and cross-validated on a sample of 100 µM ^{15}N^{13}C-labelled ubiquitin using triple resonance HNCA and HNCOCA spectra and a ^{15}N-edited NOESY-HSQC spectrum.

Expression and purification of Dss1 for NMR studies

For NMR measurements, Dss1 was expressed in *Escherichia coli BL21 (DE3)* from the pGEX6P1 vector in M9 medium containing ^{15}N ($^{15}NH_4Cl$) and ^{13}C ($^{13}C_6$-glucose) (or unlabelled) as the single sources of nitrogen and carbon. Harvested cells were lysed by sonication in a buffer containing 50 mM Tris/HCl pH 7.5, 150 mM NaCl, 10 % glycerol, and protease inhibitor tablets (Sigma). Following centrifugation at 20000 g, the cleared lysates were incubated with glutathione–Sepharose beads (GE Healthcare), washed with 50 column

volumes of 50 mM Tris/HCl pH 7.5, 150 mM NaCl and eluted with 5 column volumes 50 mM Tris/HCl, pH 8.0 containing 10 mM reduced glutathione. The eluate was buffer exchanged into 50 mM Tris/HCl pH7.5, 150 mM NaCl, 1 mM EDTA and 1 mM DTT. Subsequently, the GST-tag was cleaved with Prescission protease (Invitrogen). Prescission protease and GST-tag were removed by re-incubation with glutathione–Sepharose beads (GE Healthcare) in a buffer containing 50 mM Tris/HCl pH 7.5 and 150 mM NaCl. All samples were exchanged into 25 mM Tris/HCl pH 7.5, 50 mM NaCl prior to NMR measurements and concentrated. Unlabelled and ^{15}N,^{13}C-labelled ubiquitin (Sigma) were used for titration experiments with ^{15}N,^{13}C-labeled and unlabelled Dss1, respectively.

NMR data processing and data analyses

The X-carrier frequency was determined by referencing to internal DSS and indirectly for ^{15}N and ^{13}C dimensions using the conversion factors as described (Wishart et al., 1995). The spectra were processed using nmrDraw/nmrPipe (Delaglio et al., 1995) and qMDD (Orekhov and Jaravine, 2011). The processed spectra were analysed in CcpNmr Analysis (Vranken et al., 2005). Chemical shift perturbations (CSP) in the absence and presence of varying concentrations of ubiquitin were calculated using equation 1:

$$\Delta\delta_{obs} = \sqrt{\gamma_H(\delta H^N_{free} - \delta H^N_{obs})^2 + \gamma_N(\delta N_{free} - \delta N_{obs})^2} \quad \text{(Eq. 1)}$$

Where $\delta H_{free}{}^N$ and $\delta H_{obs}{}^N$ are the proton chemical shift in the absence and presence of ubiquitin, respectively, and $\delta_{free}{}^N$ and $\delta_{obs}{}^N$ are chemical shift in the absence and presence of ubiquitin weighted by their respective (numerical) gyromagnetic ratios, γ_H and γ_N. C^α

chemical shifts δ_{obs} were obtained from 3D HN(CO)CA spectrum and referenced to the predicted random coil chemical shifts δ_{ref} by (Kjaergaard et al., 2011) using equation 2.

$\Delta\delta = \delta_{obs} - \delta_{ref}$ (Eq. 2)

For cross-titration experiments and determination of dissociation constants, stock samples were mixed to yield the final concentrations of 25, 50, 75, 100, 125, 187.5, 250, 750, 1250 and 2500 μM ubiquitin. The change in peak intensity as a function of increasing ubiquitin concentration was fitted as described (Dagil et al., 2013).

Supplemental References

1. Cox, J., and Mann, M. (2008). MaxQuant enables high peptide identification rates, individualized p.p.b.-range mass accuracies and proteome-wide protein quantification. Nat. Biotechnol. 26, 1367-1372.

2. Cox, J., Neuhauser, N., Michalski, A., Scheltema, R.A., Olsen, J.V., and Mann, M. (2011). Andromeda: a peptide search engine integrated into the MaxQuant environment. J. Proteome. Res. 10, 1794-1805.

3. Delaglio, F., Grzesiek, S., Vuister, G.W., Zhu, G., Pfeifer, J., and Bax, A. (1995). NMRPipe: a multidimensional spectral processing system based on UNIX pipes. J. Biomol. NMR 6, 277-293.

4. Kjaergaard, M., Brander, S., and Poulsen, F.M. (2011). Random coil chemical shift for intrinsically disordered proteins: effects of temperature and pH. J. Biomol. NMR 49, 139-149.

5. Mannen, T., Andoh, T., and Tani, T. (2008). Dss1 associating with the proteasome functions in selective nuclear mRNA export in yeast. Biochem. Biophys. Res. Commun. 365, 664-671.

6. Orekhov, V.Y., and Jaravine, V.A. (2011). Analysis of non-uniformly sampled spectra with multi-dimensional decomposition. Prog. Nucl. Magn Reson. Spectrosc. 59, 271-292.

7. Shevchenko, A., Tomas, H., Havlis, J., Olsen, J.V., and Mann, M. (2006). In-gel digestion for mass spectrometric characterization of proteins and proteomes. Nat. Protoc. 1, 2856-2860.

8. Vranken, W.F., Boucher, W., Stevens, T.J., Fogh, R.H., Pajon, A., Llinas, M., Ulrich, E.L., Markley, J.L., Ionides, J., and Laue, E.D. (2005). The CCPN data model for NMR spectroscopy: development of a software pipeline. Proteins 59, 687-696.

9. Wishart, D.S., Bigam, C.G., Yao, J., Abildgaard, F., Dyson, H.J., Oldfield, E., Markley, J.L., and Sykes, B.D. (1995). 1H, 13C and 15N chemical shift referencing in biomolecular NMR. J. Biomol. NMR 6, 135-140.
